# Incidence of Infective Endocarditis Post-TPVR with MELODY Valve in Pediatric Patients: A Systematic Review and Meta-Analysis

**DOI:** 10.2174/011573403X324878240903045701

**Published:** 2024-09-16

**Authors:** Sruthi Veldurthy, Deepali Shrivastava, Farhat Majeed, Tooba Ayaz, Aqssa Munir, Ali Haider, Maneeth Mylavarapu

**Affiliations:** 1 Department of Pediatrics, Mediciti Institute of Medical Sciences, Telangana, India;; 2 Department of Anesthesia, University of Minnesota, Minneapolis, USA;; 3 Department of Medicine, Pakistan Medical and Dental Council, Islamabad, Pakistan;; 4 Department of Medicine, Baqai Medical University, Karachi, Pakistan;; 5 Department of Anesthesia, Mayo Hospital, Lahore, Pakistan;; 6 Department of Allied Health Sciences, The University of Lahore, Gujrat, Pakistan;; 7 Department of Public Health, Adelphi University, NY, USA

**Keywords:** TPVR, MELODY valve, infective endocarditis, pediatric population, heart failure, COVID-19

## Abstract

**Introduction:**

Infective Endocarditis (IE) has emerged to be one of the most impactful adverse complications post-transcatheter procedures, especially Transcatheter Pulmonary Valve Replacement (TPVR). We conducted a systematic review and meta-analysis with the aim of identifying the incidence of IE post-TPVR with the MELODY valve in the pediatric population.

**Methods:**

A comprehensive literature search was performed across several prominent databases, including PubMed/MEDLINE, SCOPUS, and Science Direct. Studies compared the clinical outcomes of pediatric patients who received TPVR using the MELODY valve. Data extraction was done for variables like the total pediatric patient population that underwent TPVR with MELODY valve, mean age, the sex of the patients, the incidence rate of IE following the procedure, and the duration between the procedure and the occurrence of IE. Inverse Variance was used to estimate the incidence of IE in patients who underwent TPVR with respective 95% confidence interval (CI).

**Results:**

In total, 4 studies with 414 pediatric patients who underwent TPVR using the MELODY valve were included in the study. The mean age of the study population was 12.7 ± 3.11 years. The pooled incidence of IE following TPVR with MELODY valve in the pediatric population was 17.70% (95% Cl 3.84-31.55; *p<*0.00001). Additionally, the mean length of duration to develop IE following TPVR with MELODY valve in the pediatric population was 2.18 years (95% Cl 0.35-4.01; *p<*0.00001).

**Conclusion:**

Our meta-analysis reveals that IE post-TPVR with MELODY valve in pediatric patients is a significant complication, clinically and statistically. Further research needs to be done to understand the risk factors and develop better management strategies.

## 1 INTRODUCTION

Infective Endocarditis (IE) has emerged to be one of the most impactful adverse complications post-transcatheter procedures, especially Transcatheter Pulmonary Valve Replacement (TPVR).

TPVR is a minimally invasive procedure used to replace a damaged or malfunctioning pulmonary valve in pediatric and adult patients born with congenital heart disease. The pulmonary valve, which is responsible for regulating blood flow from the heart's right ventricle to the lungs, can exhibit severe dysfunction, resulting in diminished blood flow or right-sided heart failure. TPVR involves the insertion of a catheter through a blood vessel, often accessed *via* the groin, and navigating it to the heart to implant a new valve, thereby avoiding the need for open heart surgery [1].

From April 2016 to March 2021, a total of 4,513 TPVR procedures were performed in patients with a median age of 19 years. Among them, 57% received the Melody (Medtronic Inc) valve, while 43% received the SAPIEN valve (Edwards Lifesciences) [1]. Although the outcomes were vastly successful, complications were seen in some patients, including but not limited to ventricular arrhythmias, Aortic root compression, post-procedure Femoral vein bleeding, and IE [2-5].

IE constitutes a significant long-term adverse outcome and remains a leading cause of mortality in patients with congenital heart disease and those who underwent TPVR [6]. McElhinney *et al*., in a multicenter study, reported that 182 of 2,476 patients were identified with endocarditis after TPVR, with a consistent hazard over time and an incidence rate of 2.2 per 100 patient-years [7].

However, IE post-TPVR, especially with the MELODY valve, was not well-researched in the pediatric population. Hence, we conducted a systematic review and meta-analysis with the aim of identifying the incidence of IE post-TPVR with the MELODY valve in the pediatric population.

## 2 METHODS

This analysis was conducted in accordance with the PRISMA guidelines (Preferred Reporting Items for Systematic Reviews and Meta-analyses) [8]. A comprehensive search was performed across several prominent databases, including PubMed/MEDLINE, SCOPUS, and Science Direct. The search strategy was formulated using appropriate Boolean operators with the following keywords: “Transcatheter Pulmonary Valve Replacement,” “MELODY Valve,” “Endocarditis,” and “Pediatric patients.” The search was limited to articles published between 2010 and 2023. The search strategy utilized for the study is outlined in **Supplementary File S1**. The references of the selected studies were also reviewed to ensure that no pertinent information was overlooked. Studies comparing the clinical outcomes of patients who received TPVR using the MELODY valve were included in our review. A detailed list of inclusion and exclusion criteria is outlined in **Supplementary File S2.** The titles and abstracts (TiAb) were screened independently by two reviewers, SV and TA, and conflicts were resolved by the third reviewer, MM. Similarly, full-text screening was independently done by two reviewers, DS and FM, and conflicts were resolved by the third reviewer, MM. Fig. (**1**) depicts the PRISMA flow chart outlining the study selection process [9].

Risk of Bias (**Supplementary File S3**) assessment was done with the help of the ROB2 tool. Data extraction was done for variables like the total pediatric patient population that underwent TPVR with MELODY valve, mean age, the sex of the patients, the incidence rate of IE following the procedure, and the duration between the procedure and the occurrence of IE. Inverse Variance was used to estimate the incidence of IE in patients who underwent TPVR with respective 95% confidence interval (CI). Analysis was performed with RevMan 5.4.1 (Cochrane, 2020). A total of I2 statistics were used to assess for heterogeneity. Funnel plots were used to assess for publication. A *p*-value ≤ 0.05 was considered statistically significant (Supplementary material).

## 3 RESULTS

**Fig. (2):**
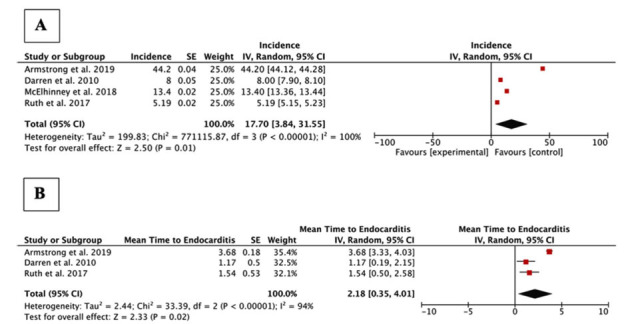
Funnel plots of infective endocarditis post-TPVR in pediatric patients. **Legend:** A. Incidence of Infective Endocarditis Post-TPVR; B. Mean Time to Infective Endocarditis Post TPVR.

A total of 4 studies were included in this meta-analysis [5, 10-12]. Of these, two were prospective [5, 10], and 2 were retrospective cohort studies [11, 12]. In total, 414 pediatric patients who received TPVR using the MELODY valve were included in the study. The mean age of the study population was 12.7 ± 3.11 years, and a majority of patients were males (70.6%) (based on three studies). The baseline characteristics of the included studies are described in Table **1**. The meta-analysis revealed that the pooled incidence of IE following TPVR with MELODY valve in the pediatric population was 17.70% (95% Cl 3.84-31.55; *p<*0.00001). Additionally, the mean length of duration to develop IE following TPVR with MELODY valve in the pediatric population was 2.18 years (95% Cl 0.35-4.01; *p<*0.00001) (Fig. **2**). Publication bias was insignificant (**Supplementary File S4**).

## 4 DISCUSSION

The results of our study revealed that out of the total pediatric population that was being investigated for IE post-TPVR with MELODY valve, 18% of the cases turned out to be positive. Furthermore, the mean time duration for the visibility of the initial symptoms of endocarditis post-TPVR is approximately two years.

Our study was the first meta-analysis to examine the incidence of IE post-TPVR with MELODY valve in pediatric patients. However, previous studies were conducted on IE post-TPVR in the adult population. Abdelghani *et al.* reported that the risk of IE post-TPVR with MELODY valve is significant, and the risk extends over three years post-procedure, validating our findings. Furthermore, Abdelghani *et al.* reported that diagnosis of IE post-TPVR with MELODY valve is often challenging [13]. Studies also reported that the risk of IE is higher in TPVR with a MELODY valve compared to Surgical Pulmonary Valve Replacement (SPVR) in the general population [13-15]. Studies have also reported that the incidence of IE post-TPVR with the SAPIEN valve is almost 80% lower compared to the MELODY valve [16].

Investigation into IE post-TPVR in pediatric patients is highly essential. An analysis of the cases of endocarditis post-TPVR would help clarify the risk factors related to IE in the pediatric population. It will also help in better understanding of the complications and enable strategic management of IE in the pediatric population. Furthermore, by investigating IE post-TPVR in pediatric patients, healthcare professionals can upgrade the diagnostic and treatment criteria to mitigate the risk of infection.

However, our study is not devoid of limitations. The primary limitation is the lack of sample size, affecting the generalizability of the findings. This further implies the lack of research on IE post-TPVR in pediatric patients. Future research with large-scale participants with rigorous methodologies needs to be done with an exclusive focus on pediatric patients who have undergone TPVR to better understand the subject and develop better management strategies.

## CONCLUSION

Our meta-analysis reveals that IE post-TPVR with MELODY valve in pediatric patients is a significant complication, clinically and statistically. Nonetheless, further research needs to be done to understand the risk factors and develop better management strategies.

## Figures and Tables

**Fig. (1):**
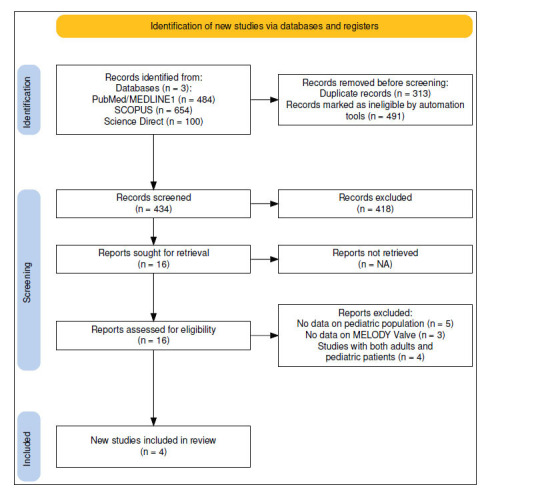
PRISMA flow chart of included studies.

**Table 1:** Baseline characteristics of included studies.

**S. No.**	**Authors**	**Year**	**Type of Study**	**Total TPVR Patients with MELODY (n)**	**Age (Mean ± SD / Median + Range)**	**Females**	**Infective Endocarditis**	
**-**	**-**	**-**	**-**	**-**	**-**		**Total I.E. with MELODY (n)**	**Time to Endocarditis (in years) (Mean ± SD)**
1	Armstrong *et al.*	2019	Prospective	156	13.25 ± 3.17	47	69	3.68 ± 1.49
2	McElhinney *et al.*	2018	Prospective	156	-	-	21	-
3	Ruth *et al.*	2017	Retrospective	77	12.97 ± 1.56	18	4	1.54 ± 1.07
4	Darren *et al.*	2010	Retrospective	25	8.45 ± 3.18	11	2	1.17 ± 0.7

## Data Availability

Not applicable.

## LIST OF ABBREVIATIONS

IE: Infective Endocarditis
TPVR: Transcatheter Pulmonary Valve Replacement
CI: Confidence Interval
